# Coagulation and Placenta-Mediated Complications

**DOI:** 10.5041/RMMJ.10168

**Published:** 2014-10-29

**Authors:** Ian A. Greer, Anat Aharon, Benjamin Brenner, Jean-Christophe Gris

**Affiliations:** 1 Faculty of Health & Life Sciences, University of Liverpool Foundation Building, Liverpool, UK;; 2 Department of Hematology and Bone Marrow Transplantation, Rambam Health Care Campus, Haifa, Israel;; 3 Bruce Rappaport Faculty of Medicine, Technion, Israel Institute of Technology, Haifa, Israel; 4 Department of Hematology, Centre Hospitalier Universitaire, Nîmes, France

**Keywords:** Placenta-mediated complications, recurrent pregnancy loss, thrombophilia

## Abstract

Pregnancy is a physiological hypercoagulable state, preparing the mother for the hemostatic challenge of delivery. However, this is associated with an increased risk of venous thrombosis and placenta-mediated complications, which present major challenges for mother and fetus. Although these conditions are heterogeneous in their pathophysiology, hereditary and acquired thrombophilia has been associated with recurrent pregnancy loss and gestational vascular complications, such as early-onset pre-eclampsia and placental abruption. Prevention of such placenta-mediated complications, which collectively complicate up to 15% of pregnancies, is a major issue for women’s health. Prospective interventional studies stratified by current knowledge of pathophysiological mechanisms related to placental and systemic hemostatic alterations will impact on the management of pregnancies at risk of these complications.

## EPIDEMIOLOGY AND PATHOGENESIS OF PREGNANCY COMPLICATIONS

Recurrent pregnancy loss and gestational vascular complications (GVC), including placental abruption, pre-eclampsia, and small-for-gestational age (SGA) pregnancies, are major gestational pathologies associated with detrimental maternal and fetal complications. These complications, affecting 5%–15% of pregnant women, are attributed to decreased trophoblast invasion and chronic hypoxia due to uteroplacental underperfusion.^1^ The risk of recurrent pre-eclampsia is about 15% after one previous event, increasing to 30% after two consecutive pregnancies complicated by pre-eclampsia.^2^ Impaired angiogenesis is reflected by the association between the clinical presentation of the disease and serum levels of soluble Flt-1 (fms-related tyrosine kinase 1) and placenta growth factor.^3^ Reduction in circulating levels of free soluble Flt-1 in women with pre-eclampsia could alleviate clinical signs of the syndrome.^4^,^5^ Placental abruption, defined as premature separation of a normally inserted placenta prior to delivery of the fetus, complicates 0.5%–1% of pregnancies and may lead to fetal death in some cases, particularly where the abruption involves more than 50% of the placenta.

## HEMOSTATIC CHANGES DURING NORMAL PREGNANCY AND GVC

Pregnancy is a physiological hypercoagulable state characterized by a progressive increase in procoagulant activity, which is maximal around term. In addition, there is a profound decrease in physiologic anticoagulants, including reduction in protein S activity and acquired activated protein C (APC) resistance.^6^,^7^ The overall fibrinolytic activity is impaired during pregnancy but returns rapidly to normal following delivery. Levels of prothrombin fragment 1+2, thrombin-antithrombin complexes, and D-dimer increase as pregnancy progresses.^8^ These changes are considered to prepare the mother for the hemostatic challenge of delivery.

Changes in the hemostatic system can lead to excessive uteroplacental thrombosis. Reduced placental perfusion due to failed vascular remodeling within the placental bed in early pregnancy is a hallmark of pre-eclampsia. Concentrations of tissue factor (TF) and free tissue factor pathway inhibitor (TFPI) are significantly increased in maternal plasma of pre-eclamptic women compared with normal pregnancy,^9^ while the TFPI-to-TF ratio is significantly lower in patients with pre-eclampsia than in normal pregnancy. The acquired changes in the physiological anticoagulant system are associated with increased thrombin generation and reduced birth-weight.^10^ Furthermore, higher maternal plasma concentrations of plasminogen activator inhibitor-1 (PAI-1) are observed in preeclampsia compared to normal pregnancy.

## THROMBOPHILIA AND FETAL LOSS

Acquired thrombophilia is associated with an increased risk of fetal loss, mainly in the first trimester, as observed in women with essential thrombocythemia. The obstetric definition of anti-phospholipid syndrome (APLS) includes fetal loss at week 10 or later and recurrent consecutive embryonic losses (before week 10). It is currently thought that TF on maternal neutrophils is critical for the pathogenesis of APL-induced fetal loss, suggesting functional linkage between complement component C5a, TF, and neutrophil activation with subsequent decidual damage and fetal wastage.^11^

### Hereditary Thrombophilias

It is well-established that thrombophilia increases the risk of thrombosis. Several reviews and meta-analyses have attempted to assess the association between thrombophilic risk factors and gestational vascular complications.^12^,^13^ The major findings of the TREATS study, which systematically reviewed the area,^13^ point toward a modest increase in the relative risk, including a higher risk for a late pregnancy loss in the third trimester; stronger associations with recurrent pregnancy loss; and a higher risk of second trimester loss than of recurrent first trimester loss for factor V Leiden (FVL) and *FII* 20210A heterozygotes. However, differences in design and conduct of the studies included in this systematic review and meta-analysis limited the conclusions. A meta-analysis of prospective cohort studies, providing a superior methodological design, demonstrated that the probability of pregnancy loss in FVL women was 52% higher, but this study had insufficient power to detect increased risks in women with *FII* 20210A polymorphism.^14^

In the absence of clear and compelling evidence linking hereditary thrombophilias to pregnancy loss, and with no proven effective intervention, routine screening of pregnant women for hereditary thrombophilia is not recommended; however, screening of women with previous complications possibly associated with thrombophilia remains a matter of debate.

## THROMBOPHILIA AND GESTATIONAL VASCULAR COMPLICATIONS

The current data regarding the association of hereditary thrombophilia and GVCs are heterogeneous, and lack systematic integration of severity and co-factors. Nonetheless, these data demonstrate modest positive odds ratios for an association. Identifying a clear association opens up the possibility of antithrombotic interventions, therefore screening of previously symptomatic women will depend on conclusive therapeutic developments in women harboring thrombophilia.

While the global risk of placental abruption was found to be significant for a heterozygous FVL or a heterozygous *FII* 20210A polymorphism,^13^ this result was not reproduced in prospective studies.^14^ An analysis of 22 publications showed an association of placental abruption with FVL and *FII* 20210A polymorphism.^15^ A recent prospective Canadian cohort study demonstrated that carriers of FVL or prothrombin gene mutation (PGM) are not at significantly increased risk of placenta-mediated complications.^16^ Thus, future collaborative efforts should focus on women with a history of severe pregnancy complications with clear definitions to create a more homogeneous study group.

### Placental Hemostasis

Tissue factor is essential for embryogenesis, angiogenesis, invasion, and implantation. While TF is not presented on cells that are in contact with systemic circulation, the placental surface is in direct contact with maternal circulation, and trophoblast cells constitutively express high levels of TF.^17^,^18^ In addition, cytotrophoblast differentiation and fusion to syncytiotrophoblasts require initiation of apoptosis and exposure of negatively charged phospholipids on their membrane surface.^19^ The need for immediate inhibition of hemorrhage in the placental intervillous spaces during gestation, labor, and delivery explains the procoagulant nature of placental trophoblast and decidual cells.

Tissue factor levels are regulated by a variety of physiological anticoagulants, including TFPI, endothelial protein C receptor, thrombomodulin, and annexin V.

Tissue factor pathway inhibitor is produced and pooled in the microvascular endothelial cells and in trophoblast cells^18^ and is highly expressed in placental tissues from the 10th week of pregnancy up to term.^20^ Low-molecular-weight heparins (LMWHs) stimulate expression, synthesis, and release of TFPI in endothelial cells and may exert their effect in pregnant women at risk for GVC, by modulating local hemostasis at the placental syncytiotrophoblast surface.^20^

### Microparticles in Normal Pregnancy and GVC

Microparticles, shed from cell membranes upon activation or apoptosis, are involved in thrombosis, inflammation, and vascular dysfunction and may play an essential role in the maternal–placental cross-talk.^21^

Microparticles obtained from healthy pregnant women demonstrate high procoagulant activity compared to non-pregnant females. The procoagulant activity further increases in microparticles of women with GVC without a change in the TF expression, but with reduction in TFPI.^22^ A hemostatic balance, manifested by the TF/TFPI ratio on microparticles originating from maternal and placental cells, could reflect normal gestational hypercoagulability as well as the vascular injury characterizing GVC pathology.^22^

Trophoblast differentiation yields substantial shedding of TF-bearing microparticles. Syncytiotrophoblast microparticles circulating in maternal blood can lead to endothelial dysfunction, monocyte stimulation, and excessive maternal inflammatory reaction.^23^

## ANTICOAGULANT THERAPY DURING GESTATION

A review of about 2,800 treated pregnancies, evaluating safety and efficacy of LMWH in pregnancy, concluded that LMWH is the anticoagulant of choice during pregnancy.^24^

### Antithrombotics for Prevention of Pregnancy Loss

The role of LMWH in the prevention of pregnancy complications remains uncertain. This possibility was stimulated by a single-center study^25^ that demonstrated, in women with thrombophilia and one previous pregnancy loss after 10 weeks of gestation, that the use of enoxaparin (40 mg daily) resulted in a significantly better live birth rate compared to patients on low-dose aspirin (86% versus 29%, respectively). The multicenter, prospective, randomized LIVE-ENOX study compared two doses of enoxaparin in women with thrombophilia and a history of pregnancy loss and did not demonstrate a difference in live birth rates between the two dosages (84% for the 40 mg/d group and 78% for the 80 mg/d group).^26^ Another study comparing enoxaparin 40 mg/d to low-dose aspirin, reported a similar live birth rate in women with unexplained pregnancy loss in whom thrombophilia was excluded.^27^ Two recent prospective multicenter randomized trials, ALIFE and SPIN, reported that antithrombotic prophylaxis did not improve pregnancy outcome in women with at least two unexplained pregnancy losses.^28^,^29^ However, thrombophilia cases were a small minority in these trials, and the majority of cases of unexplained pregnancy loss could be attributed to other causes, including aneuploidy. Therefore, the women studied would have been heterogeneous in terms of the underlying cause of the loss such that antithrombotic treatment would have been unlikely to have been beneficial in the absence of a thrombotic mechanism for their loss.

### Antithrombotics for Prevention of Gestational Vascular Complications

A Canadian randomized trial compared dalteparin 5,000 units/d versus no prophylaxis in 110 women without identifiable thrombophilia who had prior placental-mediated complications.^30^

Dalteparin was associated with a lower rate of composite primary outcome (severe pre-eclampsia, birth-weight less than the 5th percentile, major placental abruption) which occurred in 5.5% of those in the dalteparin arm compared to 23.6% in the no prophylaxis arm (OR 0.15, 95% CI 0.03–0.70, *P* = 0.016). A single-center randomized study^31^ in women with a prior placental abruption demonstrated that prophylaxis with enoxaparin significantly reduced the primary composite outcome (31.3% versus 12.5%). Subsequent studies will be required to confirm these findings.

The recently published HAPPY trial, which had heterogeneous inclusion criteria,^32^ found no difference between intervention with LMWH (nadroparin) and the non-interventional arm for pregnancy outcome. Potential limitations of this negative trial included under-representation of severe preeclampsia, lack of information on the gestational age at the previous and present occurrence of preeclampsia, and on the number of cases of preeclampsia associated with fetal growth restriction/small for gestational age (FGR/SGA).^33^

The FRUIT study of women with heritable thrombophilia and early-onset pre-eclampsia demonstrated that adding LMWH to low-dose aspirin reduced recurrent pre-eclampsia onset before 34 weeks of gestation, but with no impact on general recurrence rates or overall outcome irrespective of gestational age.^34^

A recent Cochrane review^35^ of 14 studies assessed treatment with heparin for women considered to be at particularly high risk of pregnancy complications secondary to placental insufficiency. This review found an association with a statistically significant reduction in risk of perinatal mortality (RR 0.40, 95% CI 0.20–0.78), preterm birth before 34 (RR 0.46, 95% CI 0.29–0.73) and 37 weeks of gestation (RR 0.72, 95% CI 0.58–0.90), and birth-weight below the 10th centile for gestational age (RR 0.41, 95% CI 0.27–0.61), when compared with no treatment. A recent meta-analysis of LMWH for prevention of recurrent placenta-mediated pregnancy complications (PMPCs) performed in six randomized controlled studies which had included 848 pregnant women with prior PMPCs concluded that LMWH may be a promising therapy for secondary prophylaxis, especially for severe or early pre-eclampsia (RR 0.16, 95% CI 0.07–0.36).^36^

Recently, the TIPPS trial—a multicenter open-label randomized study evaluating an antepartum prophylactic dose of dalteparin versus no dalteparin in women with increased-risk pregnancy and thrombophilia—showed no benefit for the intervention arm.^37^ Of note, this study is characterized by a large variety of indications and a small number of patients at high risk due to previous severe placenta-mediated complications, likely making the study population heterogeneous for the disease mechanism.

## FUTURE PERSPECTIVES

Currently, we lack data on genome-wide association study (GWAS), biomarkers, and interventions applied to specific homogeneous populations. Defining and targeting specific disease processes amenable to antithrombotics and measuring the biological response as well as the clinical outcome will be valuable in advancing this field. Future studies should focus on examining antithrombotic interventions versus placebo (or suitable control) in more homogeneous populations such as thrombophilic women with well-characterized severe pregnancy complications, or those stratified by pathological findings, such as a severely infarcted placenta or validated biomarkers of the disease process.

In summary, prevention of placenta-mediated complications remains a major health issue. Prospective interventional studies based on data on the pathophysiological mechanisms and systemic hemostatic alterations will pave the way for a better management of women and fetuses at risk.

## Abbreviations:

APC: activated protein C;
APLS: anti-phospholipid syndrome;
FGR: fetal growth restriction;
Flt-1: fms-related tyrosine kinase 1;
FVL: factor V Leiden;
GVC: gestational vascular complications;
GWAS: genome-wide association study;
LMWH: low-molecular-weight heparins;
PAI-1: plasminogen activator inhibitor-1;
PGM: prothrombin gene mutation;
SGA: small for gestational age;
TF: tissue factor;
TFPI: tissue factor pathway inhibitor.
